# Burden of lower respiratory infections in five East Asian countries from 1990 to 2021: observation, comparison, and forecast from the global burden of disease study 2021

**DOI:** 10.3389/fpubh.2025.1679714

**Published:** 2025-10-24

**Authors:** Shiwei Wang, Shuai Lin, Guodong Zhong, Zhangyang Qi, Wei Wang, Wen Wen

**Affiliations:** ^1^Department of Pulmonary and Critical Care Medicine, Fuzong Clinical Medical College of Fujian Medical University, Fuzhou, Fujian, China; ^2^The Second Affiliated Hospital of Fujian University of Traditional Chinese Medicine, Fuzhou, Fujian, China; ^3^Zhongshan Hospital of Traditional Chinese Medicine Affiliated to Guangzhou University of Traditional Chinese Medicine, Zhongshan, Guangdong, China; ^4^School of Medicine, Dongfang Hospital of Xiamen University, Xiamen University, Xiamen, Fujian, China; ^5^900th Hospital of PLA Joint Logistic Support Force, Fuzhou, Fujian, China

**Keywords:** lower respiratory infections, global burden of disease, East Asia, disability-adjusted life years, temporal trends

## Abstract

**Objective:**

Lower respiratory infections (LRIs) remain a major global health challenge. Although the overall burden has declined, cross-country differences in long-term trends, age–sex patterns, and risk factors in East Asia are not well characterized. This study provides the first systematic comparison of long-term LRI trends across five East Asian countries—China, Japan, Republic of Korea, Democratic People’s Republic of Korea, and Mongolia—using Global Burden of Disease Study 2021 (GBD 2021) data.

**Methods:**

We analyzed LRI incidence, mortality, and disability-adjusted life years (DALYs) from 1990 to 2021. Temporal trends were quantified by estimated annual percentage change (EAPC). We combined decomposition analysis and autoregressive integrated moving average (ARIMA) forecasting to quantify the relative contributions of population growth, aging, and epidemiological changes, and to project future trajectories. Risk factor attribution was evaluated using population-attributable fractions, and age–sex patterns were compared across countries.

**Results:**

From 1990 to 2021, all five countries showed substantial reductions in age-standardized incidence and mortality, with Mongolia and China achieving the steepest declines. The burden shifted from children under five to older adults, particularly those aged ≥70 years, a novel epidemiological transition most evident in Japan and Korea. Decomposition indicated that epidemiological improvements were the primary drivers of mortality reductions, while population aging in Japan and Korea partially counteracted these improvements. Forecasts suggest continued declines in incidence and mortality across most countries, though Japan may experience a plateau in mortality.

**Conclusion:**

This study provides the first systematic comparison of long-term LRI trends across five East Asian countries. While the overall burden has declined, population aging, air pollution, and smoking are emerging challenges. By combining decomposition and ARIMA forecasting, our findings highlight the shift from children to older adults and offer timely evidence for age-sensitive, country-specific interventions such as vaccination for older adults, environmental regulation, and tobacco control.

## Introduction

Lower respiratory infections (LRIs) remain a leading cause of morbidity and mortality worldwide, posing a persistent public health challenge despite major advances in vaccination, nutrition, and healthcare access over the past three decades (1–4). In 2021, LRIs accounted for approximately 2.2 million deaths, ranking as the seventh leading cause of death globally (1). Although age-standardized mortality and disability-adjusted life years (DALYs) have declined since 1990, the pace of reduction has been uneven: children in low-SDI regions continue to bear disproportionate burdens, while declines have slowed—or even reversed—among older adults in high-SDI settings (2, 5). These patterns highlight LRIs as a continuing threat that is evolving alongside demographic and epidemiological transitions.

The Global Burden of Disease (GBD) framework provides a standardized platform to quantify health loss across diseases, populations, and risk factors. The 2021 iteration, the first major update after the COVID-19 pandemic, synthesizes over 328,000 data sources covering 371 diseases and 87 risk factors across 204 countries (3). By combining composite indicators such as DALYs with socio-demographic index (SDI) stratification, GBD enables robust cross-national comparisons (6). At the same time, estimates for data-scarce settings—such as the Democratic People’s Republic of Korea (DPRK)—rely heavily on predictive modeling, yielding wide uncertainty intervals (7). These methodological features underscore both the utility and limitations of GBD data when evaluating disease burden at national and subregional levels.

East Asia—comprising China, Japan, Republic of Korea, DPRK, and Mongolia—provides a compelling case for comparative analysis. Over the past three decades, these countries have undergone rapid demographic, economic, and environmental transitions but have followed divergent trajectories in healthcare infrastructure and risk exposures. Japan and Korea face advanced population aging, with older adults now accounting for a growing share of LRI deaths (2, 8). Industrialization and urbanization have intensified ambient PM2.5 exposure, while tobacco use remains widespread, particularly among older men (9, 10). Meanwhile, non-pharmaceutical interventions during the COVID-19 pandemic (e.g., masking, distancing) temporarily suppressed viral LRIs, demonstrating how behavioral and policy shifts can reshape respiratory disease epidemiology (3). Despite these dynamic changes, prior GBD analyses have offered mainly global or regional summaries without systematically comparing East Asian countries. This leaves an important gap regarding how differing demographic transitions, environmental risks, and health systems have shaped LRI trends across this region.

Against this background, the present study uses GBD 2021 data to conduct the first systematic comparison of long-term LRI trends across five East Asian countries from 1990 to 2021. We aimed to (i) characterize temporal trends in incidence, mortality, and DALYs; (ii) compare age- and sex-specific distributions and risk factor structures; and (iii) forecast trajectories through 2030 by integrating decomposition analysis with ARIMA forecasting. Our findings further reveal a novel epidemiological shift of LRI burden from children to older adults, especially in rapidly aging countries such as Japan and Korea. These contributions provide timely evidence to inform region-specific prevention strategies and global efforts to mitigate LRI burden in the context of aging, antimicrobial resistance, and climate change.

## Methods

### Overview

We systematically assessed and compared the burden of lower respiratory infections (LRIs) globally and in five East Asian countries—China, Japan, Republic of Korea, Democratic People’s Republic of Korea (DPR Korea), and Mongolia—from 1990 to 2021, using data from the Global Burden of Disease Study 2021 (GBD 2021). Four primary metrics were evaluated: incidence, prevalence, mortality, and disability-adjusted life years (DALYs). We further analyzed temporal trends, age–sex-specific patterns, risk factor attribution, and projected future trends. To disentangle the drivers of burden change, we conducted decomposition analysis to quantify the relative contributions of population growth, population aging, and epidemiological changes.

### Data sources

All data were obtained from the Global Burden of Disease Study 2021 (GBD 2021) Results, published by the Institute for Health Metrics and Evaluation (IHME), Seattle, United States, in 2022. Standardized estimates are available for 371 diseases and 87 risk factors across 204 countries and territories (11). Data were extracted via the GBD Results Tool (https://vizhub.healthdata.org/gbd-results/) from the Global Health Data Exchange (GHDx) on February 7, 2025. For each country and globally, we retrieved age-standardized rates (ASRs) of prevalence, incidence, mortality, and disability-adjusted life years (DALYs) attributable to LRIs between 1990 and 2021. All estimates were stratified by age and sex, with 95% uncertainty intervals (UIs).

### Measures of disease burden

LRI burden was assessed using four core indicators: (1) incidence rate, defined as the number of new LRI cases per 100,000 population per year; (2) prevalence rate, representing the number of existing LRI cases per 100,000 population; (3) mortality rate, defined as the number of LRI-attributable deaths per 100,000 population; and (4) DALYs, calculated as the sum of years of life lost (YLLs) due to premature death and years lived with disability (YLDs). All estimates were age-standardized using the GBD global standard population to minimize the effects of demographic differences.

### Temporal trend analysis

Temporal changes in LRI burden were assessed by extracting Estimated Annual Percentage Changes (EAPCs) of ASRs for incidence, mortality, and DALYs, as computed by the Institute for Health Metrics and Evaluation (IHME). This standardized metric quantifies the direction and magnitude of long-term changes. To enhance interpretability, we also calculated and plotted percent change in ASRs between 1990 and 2021, stratified by country, age, and sex.

### Risk factor attribution

We assessed the contributions of risk factors to LRI mortality using population attributable fractions (PAFs) from GBD 2021. In the GBD framework, risk factors are grouped into three broad categories: (i) Environmental and occupational risks – including ambient particulate matter pollution, household air pollution from solid fuels, non-optimal temperature, and unsafe water, sanitation, and handwashing. (ii) Behavioral risks – an umbrella category that encompasses tobacco use (smoking and secondhand smoke), alcohol use, drug use, dietary risks, low physical activity, unsafe sex, and other behavioral exposures. In GBD reporting, tobacco use is often displayed both individually and as part of this broader category. (iii) Metabolic and nutritional risks – including child wasting, low birth weight, suboptimal breastfeeding, and other indicators of child and maternal malnutrition. Temporal changes in the proportional contribution of these risk factors from 1990 to 2021 were analyzed to illustrate evolving epidemiological patterns across countries. Percent contributions were derived from GBD’s comparative risk assessment framework using population attributable fractions (PAFs), and the risk factor analysis was descriptive within the GBD framework rather than based on additional model fitting.

### Decomposition analysis

We applied Das Gupta’s decomposition method to assess the individual contributions of population growth, population aging, and epidemiological changes to LRI burden globally and across the five East Asian countries (12). This method was applied to incidence and mortality data for each East Asian country and globally, enabling quantitative assessment of the relative weight of demographic vs. epidemiological drivers.

### Forecasting analysis

To forecast LRI incidence and mortality trends from 2022 to 2030, we employed autoregressive integrated moving average (ARIMA) models. Stationarity was assessed using the Kwiatkowski–Phillips–Schmidt–Shin (KPSS) test, with differencing applied as required. ARIMA parameters (p, d, q) were selected based on minimum AIC/BIC values and diagnostic adequacy tests; details for each country are in Supplementary Table S3. Residual adequacy was further confirmed using Q–Q plots for normality and Ljung–Box tests for independence. This framework ensured robust short-term forecasting while accounting for serial correlation in time-series data. Models were fitted on annual age-standardized incidence rates (ASIR) and age-standardized mortality rates (ASMR) for each country.

### Statistical software

All data analyses and visualizations were conducted using R software (version 4.4.1). ARIMA modeling was performed using the “forecast” package in R (version 8.23.0). All statistical tests were two-sided, and statistical significance was defined as *p* < 0.05.

### Ethical approval

This study was based entirely on publicly available data from the GBD 2021 database and did not involve any individual-level or personally identifiable information. Therefore, institutional ethical approval and informed consent were not required.

## Results

### Current burden of LRIs in global and five East Asian countries

**Figure 1:**
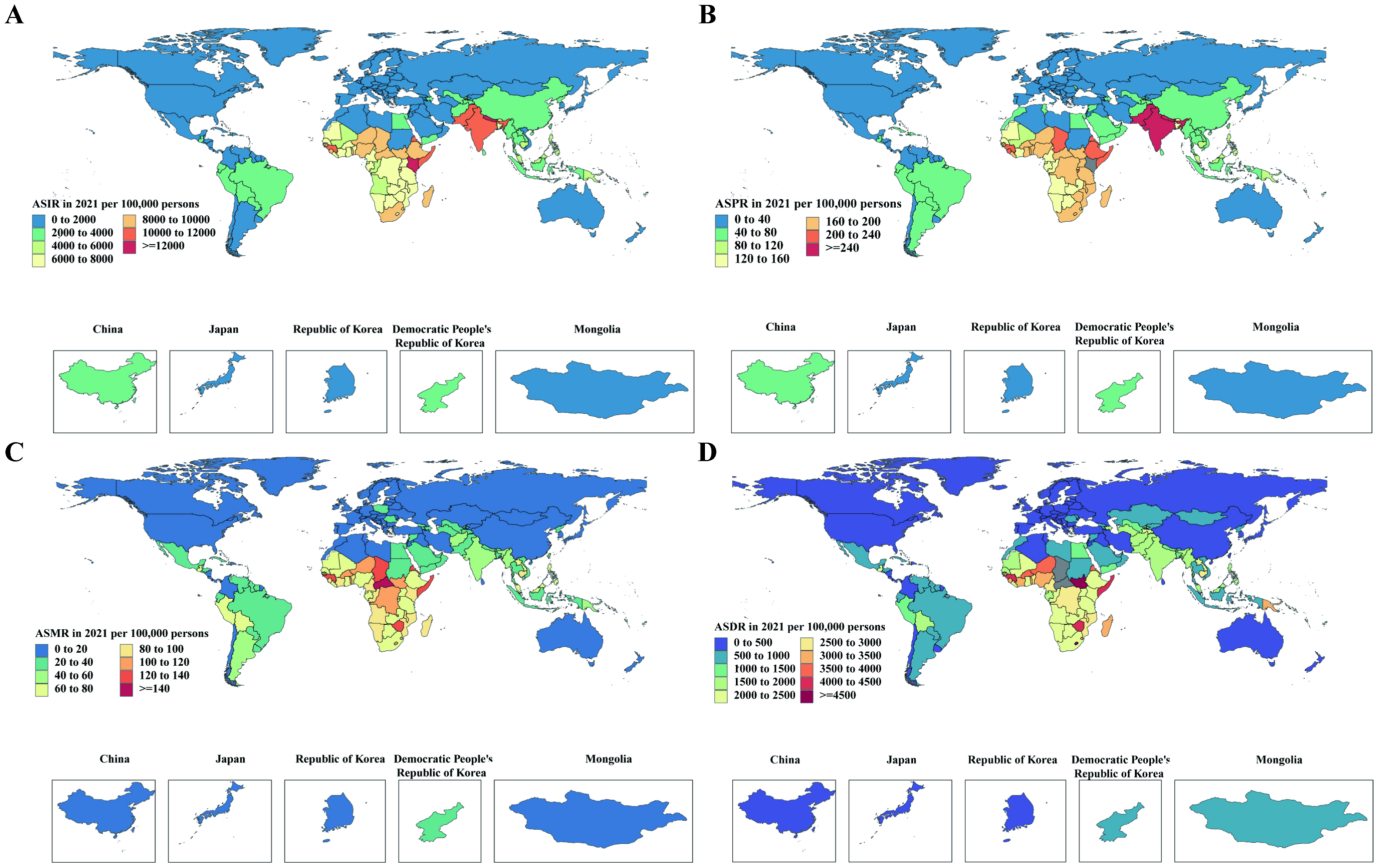
Global age-standardized incidence, prevalence, death, and DALY rates of lower respiratory infections in 2021. **(A)** Incidence rates per 100,000 population; **(B)** Prevalence rates per 100,000 population; **(C)** Death rates per 100,000 population; **(D)** DALY rates per 100,000 population. Regions with highest incidence and prevalence (>8,000 per 100,000) and mortality (>80 per 100,000) with DALYs (>2,400 per 100,000) were mainly in Sub-Saharan Africa and South Asia. East Asia and other high-income regions showed comparatively lower burdens. DALYs, Disability-Adjusted Life Years.

In 2021, substantial geographic disparities in the burden of lower respiratory infections (LRIs) were observed globally and across five East Asian countries (China, Japan, Republic of Korea, DPR Korea, and Mongolia). While large parts of sub-Saharan Africa and South Asia carried the heaviest burdens globally, with incidence, mortality, and DALYs far exceeding the global average, all five East Asian countries exhibited significantly lower rates across all metrics, underscoring their relative success in disease control and prevention (Figure 1).

### Temporal trends and age–sex patterns (1990–2021)

**Figure 2:**
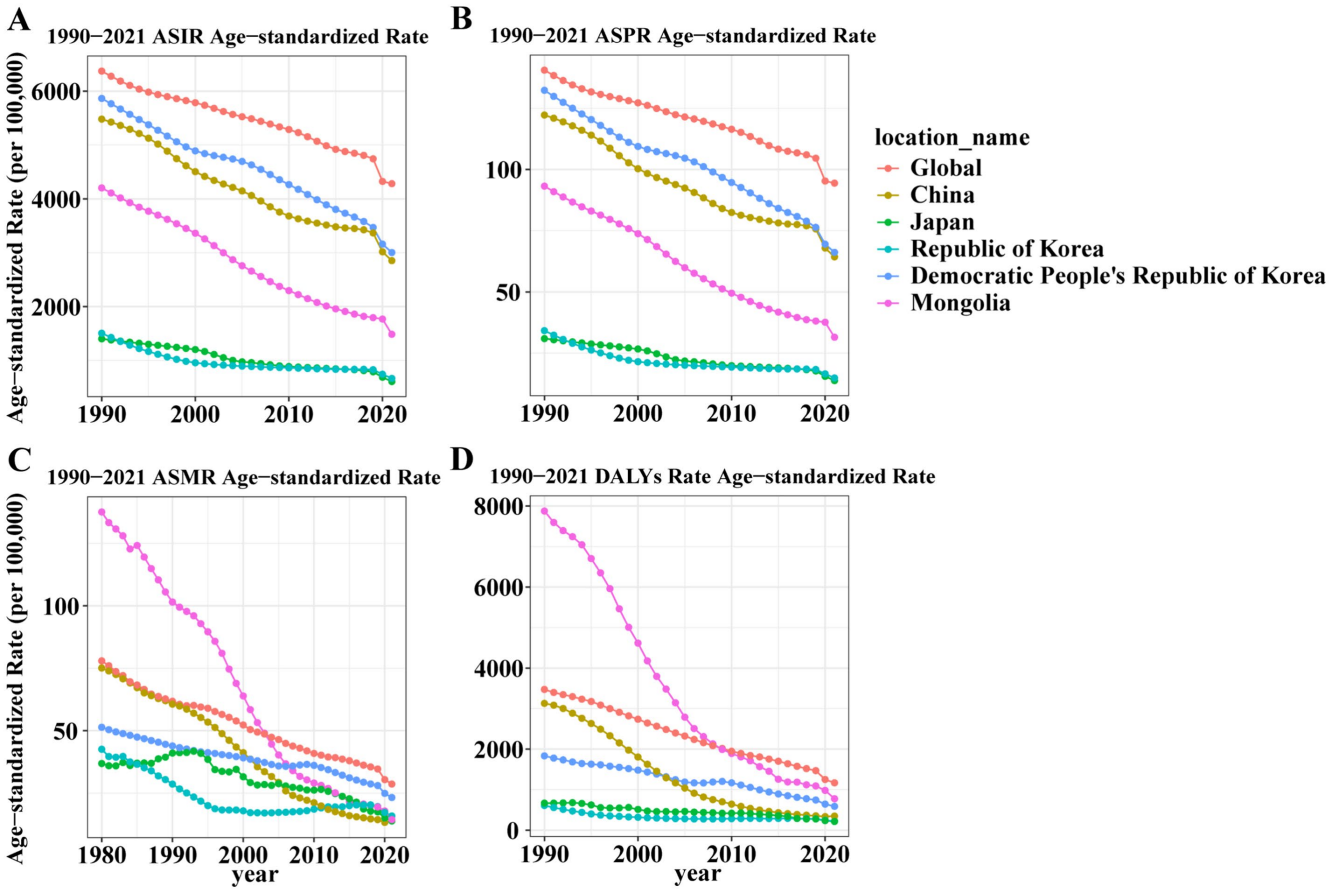
Trends of age-standardized incidence, prevalence, death, and DALY rates for lower respiratory infections globally and in Five East Asian Countries, 1990–2021. **(A)** ASIR trends; **(B)** ASPR trends; **(C)** ASMR trends; **(D)** ASDR trends. From 1990 to 2021, global and East Asian ASIR, ASPR, ASMR, and ASDR all showed consistent declines. Mongolia showed a sharp decrease in DALY rates (from ~7,000 to <1,000 per 100,000). ASIR, age-standardized incidence rate; ASPR, age-standardized prevalence rate; ASMR, age-standardized mortality rate; ASDR, age-standardized DALY rate; DALYs, Disability-Adjusted Life Years.

Between 1990 and 2021, the global LRI burden declined substantially, with ASIR decreasing by 32.8%, ASMR by 53.6%, and ASDR by 66.3% (Supplementary Table S1; Figure 2). Similar declines were observed in East Asia, though with notable variation. Mongolia achieved the steepest reductions in DALYs (−90.1%), followed by China, where ASMR and ASDR fell by 76.9 and 88.9%, respectively. Japan consistently had the lowest burden across all metrics throughout the study period.

**Figure 3:**
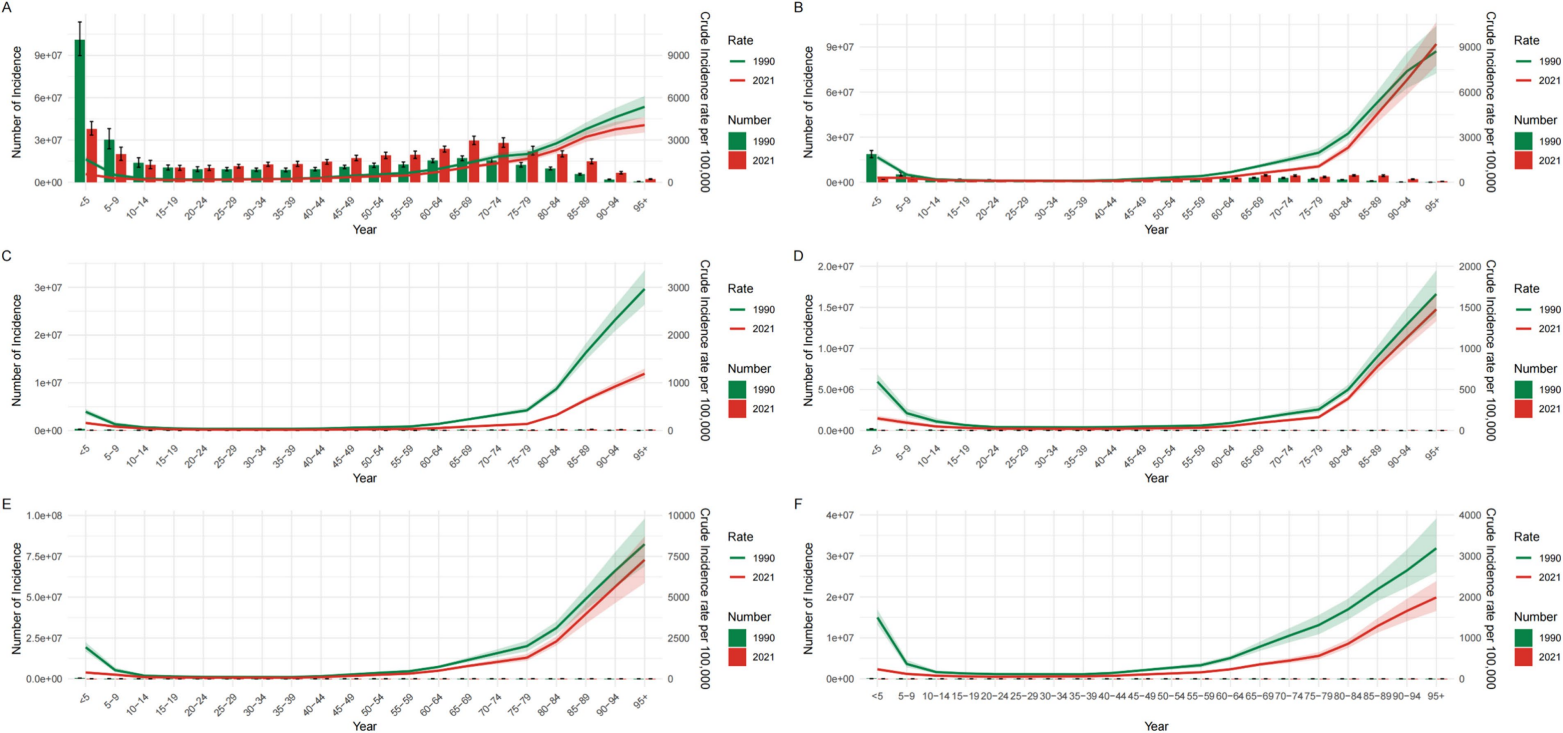
Incidence numbers and crude incidence rates of lower respiratory infections by age group globally and in Five East Asian Countries, 1990 and 2021. **(A)** Global; **(B)** China; **(C)** Japan; **(D)** Republic of Korea; **(E)** Democratic People’s Republic of Korea; **(F)** Mongolia. Incident case numbers generally increased with advancing age in both 1990 and 2021. Crude incidence rates declined substantially in children <5 years but rose in older adults (≥70 years). Country panels show similar age shifts, with Mongolia retaining higher child incidence and notable older population increases by 2021. CIR, Crude Incidence Rate.

**Figure 4:**
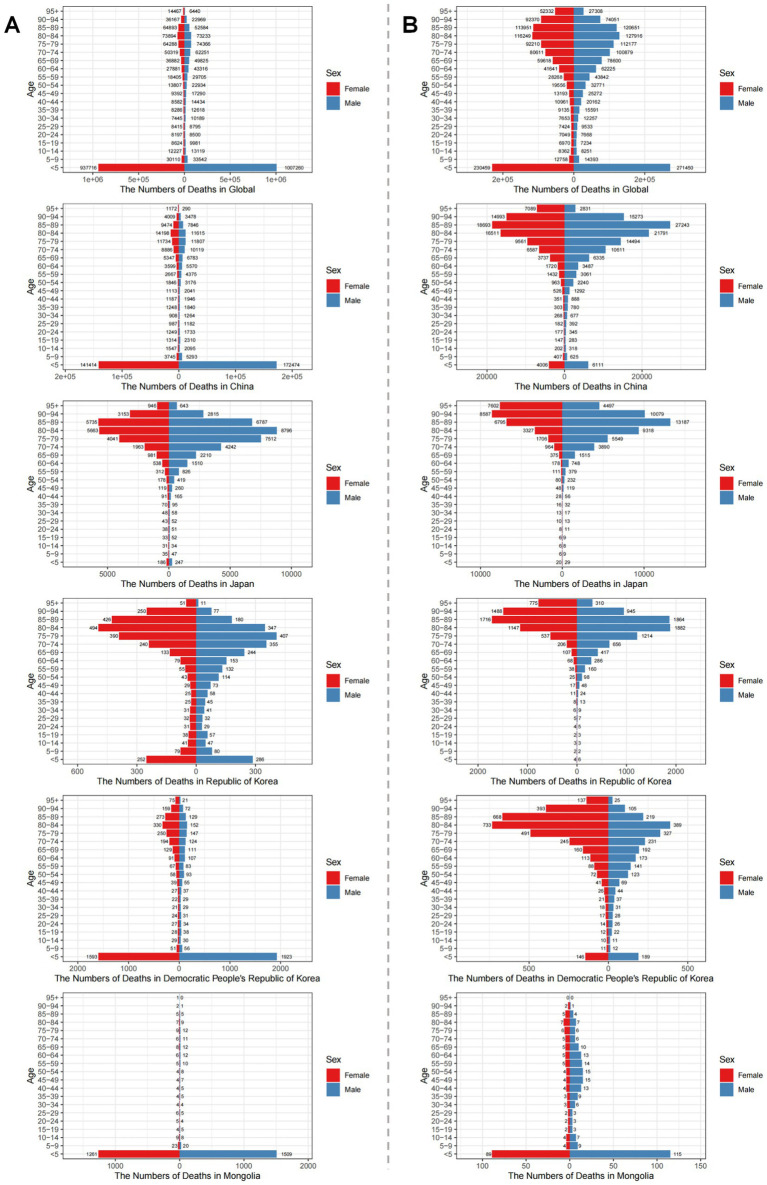
Age-sex distribution of death numbers due to lower respiratory infections globally and in Five East Asian Countries in 1990 and 2021. **(A)** Death numbers in 1990; **(B)** Death numbers in 2021. In 1990, LRI deaths were concentrated among children <5 years, but by 2021 shifted toward older populations (≥70 years) globally and across East Asian countries. Panel comparisons illustrate the magnitude of this age shift by sex and country.

From 1990 to 2021, the age distribution of LRI burden shifted profoundly from a predominantly pediatric to a geriatric profile (Supplementary Figures S1, S2; Figures 3, 4). In 1990, incidence and mortality were concentrated among children under five globally and in most East Asian countries, reflecting the classic pediatric pattern of LRIs. By 2021, however, childhood rates had declined substantially, while incidence and deaths increasingly clustered among adults ≥60 years, especially those ≥70. This transition was most pronounced in Japan and the Republic of Korea, where older populations already bore a large share of the burden in 1990 and showed further intensification by 2021. In China, sharp reductions in under-five mortality were offset by rising absolute deaths among older adults due to rapid population aging. In contrast, Mongolia and DPR Korea retained higher burdens in young children but also experienced notable increases among the older adults, indicating a dual burden. Across most countries, incidence patterns were broadly similar between sexes, though mortality showed important differences: in 2021, female mortality in DPR Korea significantly exceeded that of males, pointing to context-specific gender disparities. Collectively, these findings highlight a structural migration of LRI burden from children to older adults, with cross-country heterogeneity shaped by demographic aging, healthcare access, and sociopolitical context.

### Decomposition of drivers of change

Decomposition analysis quantified the contributions of population growth, aging, and epidemiological changes to shifts in LRI burden between 1990 and 2021 (Supplementary Table S2; Supplementary Figure S3). Globally, incidence increases were mainly driven by population growth, while mortality declines reflected epidemiological improvements partly offset by aging. In East Asia, a similar pattern was observed, though with notable variation: China and Mongolia benefited most from epidemiological gains, whereas aging exerted stronger counteracting effects in Japan and the Republic of Korea. DPR Korea showed mixed results, with aging contributing substantially to mortality despite progress in other domains. Sex-stratified analyses revealed consistent trends, though the impact of aging was slightly stronger among females in China and Korea. Collectively, these findings indicate that while epidemiological progress has been the dominant driver of LRI reduction, population aging is increasingly eroding gains in higher-income East Asian countries.

### Risk factor contributions in 2021

**Figure 5:**
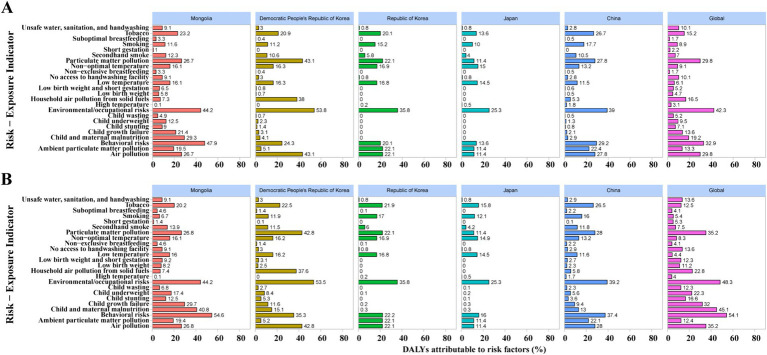
Risk factors contributing to LRI-related deaths **(A)** and DALYs **(B)** in 2021 in Mongolia, Democratic People’s Republic of Korea, Republic of Korea, Japan, China, and globally. **(A)** The percentage of deaths attributable to individual risk factors; **(B)** the corresponding percentages for disability-adjusted life years (DALYs).

In 2021, environmental and behavioral risks were the dominant drivers of LRI-related deaths and DALYs across East Asia, with notable cross-country variation (Figure 5). Globally, air pollution and behavioral risks—largely tobacco use—were the main contributors, while child and maternal malnutrition remained important in low-SDI settings. Within East Asia, China’s burden was driven mainly by ambient particulate matter and smoking, Japan and the Republic of Korea by tobacco use (especially among older men), and Mongolia and DPR Korea by a dual burden of rising environmental and behavioral risks alongside persistent child undernutrition. These contrasts underscore distinct stages of epidemiological transition across the region.

### Forecasting trends in LRI burden (2022–2030)

**Figure 6:**
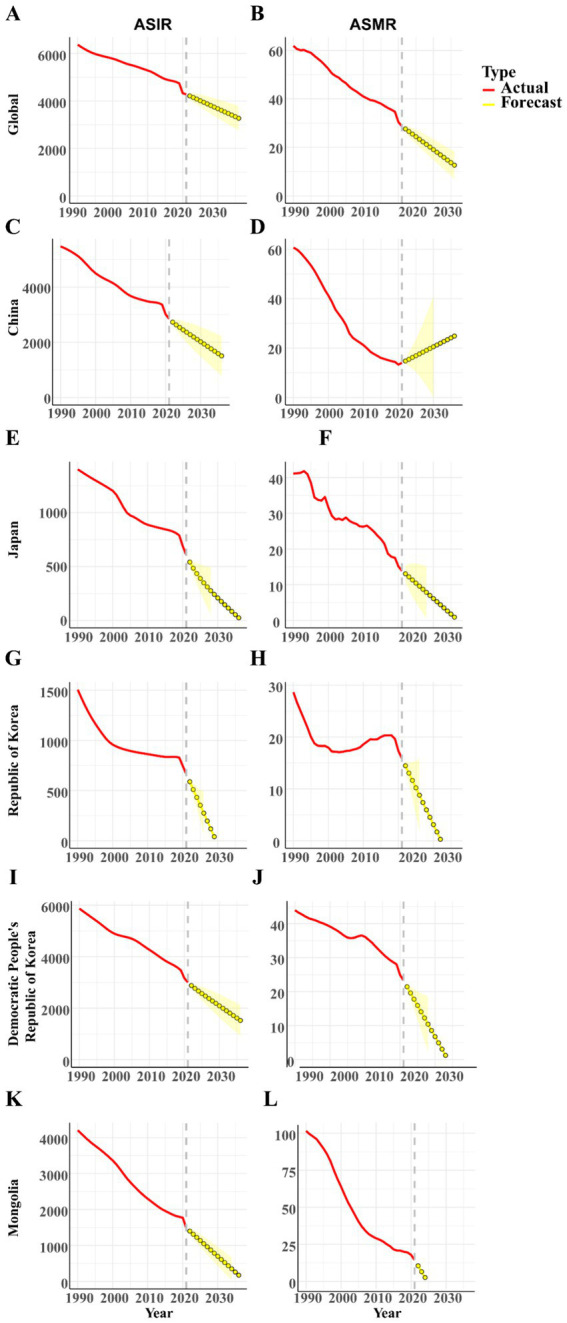
Forecasted trends of age-standardized incidence and mortality rates of lower respiratory infections globally and in Five East Asian Countries, from 2022 to 2030. **(A,B)** Global incidence and mortality rates; **(C,D)** Incidence and mortality rates in China; **(E,F)** Incidence and mortality rates in Japan; **(G,H)** Incidence and mortality rates in Republic of Korea; **(I,J)** Incidence and mortality rates in Democratic People’s Republic of Korea; **(K,L)** Incidence and mortality rates in Mongolia. ARIMA-forecasted trends in age-standardized incidence and mortality of LRIs globally and in five East Asian countries, 2022–2030. Panels show country-specific trajectories with 95% forecast intervals. ASIR, age-standardized incidence rate; ASMR, age-standardized mortality rate; LRI, lower respiratory infection.

ARIMA forecasts suggest that both global and regional burdens of LRIs will continue to decline through 2030, albeit with heterogeneity across countries (Figure 6). At the global level, ASIR are projected to decrease by approximately 12% (Figure 6A), while ASMR are expected to fall by nearly 20% (Figure 6B). In East Asia, the Democratic People’s Republic of Korea (Figures 6I,J) and Mongolia (Figures 6K,L) are forecasted to experience the steepest relative declines, with LRI mortality decreasing by more than one-third, reflecting rapid epidemiological improvements. China (Figures 6C,D) and the Republic of Korea (Figures 6G,H) are projected to show more moderate reductions in both incidence and mortality. In Japan (Figures 6E,F), projected mortality declines are minimal, and the 95% forecast band encompasses a near-flat trajectory beyond 2025, indicating limited additional gains under status-quo conditions.

Together, these forecasts highlight that although the overall burden of LRIs is expected to continue decreasing, the pace and magnitude of improvement will vary considerably across countries, underscoring the need for tailored, context-specific strategies to sustain progress in respiratory health.

## Discussion

### Epidemiological gains vs. population aging

Our analysis demonstrates a substantial decline in LRI burden across East Asia over the past three decades, mirroring global trends yet with distinct regional features. Age-standardized incidence, mortality, and DALY rates fell markedly in China, Japan, Republic of Korea, DPRK, and Mongolia, with China and Mongolia achieving particularly steep reductions. These gains likely reflect expanded vaccine coverage, improved healthcare access, and environmental control policies (13–15). However, decomposition analysis shows that population aging and growth have partially offset these improvements, shifting the burden from children to older adults. This shift carries important clinical implications, as older adults are more vulnerable to LRIs due to immunosenescence, multimorbidity, and suboptimal vaccine uptake (16, 17). As a result, LRIs can no longer be regarded as primarily a pediatric condition but are becoming a geriatric challenge, requiring older adult-focused preventive strategies and health system adaptation.

Notably, Japan and Korea already exhibited high burden in people over 65 in 1990, and this intensified further by 2021, reflecting advanced demographic aging. In China, sharp reductions in under-five mortality were offset by rising absolute deaths among older adults due to rapid population aging. Mongolia and DPRK, meanwhile, retain higher burdens in young children but also show increasing incidence and mortality among the older populations, creating a dual burden. Together, these findings highlight the tension between epidemiological progress and demographic aging, which is reshaping the clinical and public health profile of LRIs across East Asia.

### Cross-country risk factor structures

Risk factor attribution further refines the policy agenda. Environmental exposures, especially ambient particulate matter, now rival or surpass traditional risks such as household air pollution and malnutrition (18, 19). In China, although mortality has declined, DALYs attributable to PM2.5 and smoking have increased, reflecting a shift toward environment- and lifestyle-related drivers. In Japan and Korea, smoking among older men remains the leading contributor (10). In Mongolia and DPRK, a dual burden persists: environmental and behavioral risks are rising, while nutrition-related factors—particularly child growth failure and underweight—still account for a considerable share of LRI burden.

These cross-country contrasts underscore that strategies must be tailored to demographic and epidemiological contexts. For example, stricter air quality control and clean energy adoption are priorities in China and Mongolia; intensified tobacco control is critical in Japan and Korea; while strengthened vaccination, nutrition, and primary care remain essential in DPRK and Mongolia. Globally, malnutrition continues to dominate DALY losses in low-SDI regions, reminding us that East Asia’s epidemiological transition should not obscure unfinished challenges. Targeted measures aligned with national drivers will yield greater impact than broad, generic interventions (20, 21).

### Policy implications of forecasts

ARIMA forecasts suggest that LRI burdens will continue to decline through 2030, though with heterogeneous trajectories. DPRK and Mongolia are projected to achieve the steepest reductions, reflecting the benefits of strengthened vaccination and health system improvements. China and Korea show moderate declines, underscoring the need to sustain progress through tighter air quality control and tobacco reduction. By contrast, Japan exhibits only marginal decreases in mortality after 2025, with projections approaching a plateau. This near-flat trend is plausible given Japan’s super-aging population and the high prevalence of aspiration pneumonia in older adults, a condition less responsive to vaccine-based interventions (22). These findings highlight the need for strategies targeting older populations, such as aspiration pneumonia prevention, geriatric care integration, and adult immunization programs.

At the same time, external shocks could alter expected trajectories. Antimicrobial resistance is projected to cause millions of deaths annually by 2050, with respiratory pathogens central to this burden (23, 24). Climate change may exacerbate risks through rising temperatures, pollution, and altered viral seasonality (25, 26). The COVID-19 pandemic further illustrates how NPIs temporarily suppressed LRIs (27, 28), but also introduced uncertainties such as “immunity debt” in children and vulnerability among older adults due to disrupted healthcare (29, 30). Together, these insights emphasize that reliance on past trends alone may underestimate future risks. To sustain progress, countries must pair ongoing preventive measures with robust pathogen surveillance, AMR stewardship, and age-sensitive, context-specific health policies.

### Limitations

This study has several limitations. First, GBD estimates are constrained by data availability; in DPRK and Mongolia, reliance on modeled inputs increases uncertainty. Second, GBD 2021 data extend only through 2021, excluding later phases of the COVID-19 pandemic, which may have had further effects on LRI burden. The pandemic represents a critical inflection point, with possible misclassification of mortality data and uncertainties such as immunity debt in children and heightened vulnerability among older adults. Third, ARIMA models assume continuity of past trends and may not fully capture disruptive events such as pandemics, vaccination campaigns, or climate shocks. Forecasts are inherently model-based and sensitive to demographic shifts; thus, apparent plateaus—such as those projected for Japan—should be interpreted cautiously and validated with post-2021 data. Finally, we acknowledge that our analytical framework primarily applied established approaches. Future research could incorporate more innovative forecasting techniques—such as Bayesian hierarchical models or machine learning–based approaches—and integrate multi-source surveillance data to further improve predictive accuracy and causal inference.

## Conclusion

In summary, the LRI burden in East Asia has declined substantially, but the demographic and epidemiological transition toward older populations, coupled with persistent environmental and behavioral risks, demands recalibrated strategies. Country-specific, age-sensitive interventions—ranging from air quality control to adult immunization and AMR preparedness—are essential to sustain progress. By situating national trends within broader global challenges, our findings underscore the need for adaptive, evidence-based policies to address the next phase of respiratory infection control in the era of aging, urbanization, environmental change, and pandemic preparedness.

## Data Availability

Publicly available datasets were analyzed in this study. This data can be found here: (https://vizhub.healthdata.org/gbd-results/).
