# Supplementary material for: Burden of lower respiratory infections in five East Asian countries from 1990 to 2021: observation, comparison, and forecast from the global burden of disease study 2021
Source: Front Public Health. 2025 Oct 24;13:1679714. doi: 10.3389/fpubh.2025.1679714 (PMC12592797; doi:10.3389/fpubh.2025.1679714)
Supplement: Supplementary file 1 [file Data_Sheet_1.pdf]

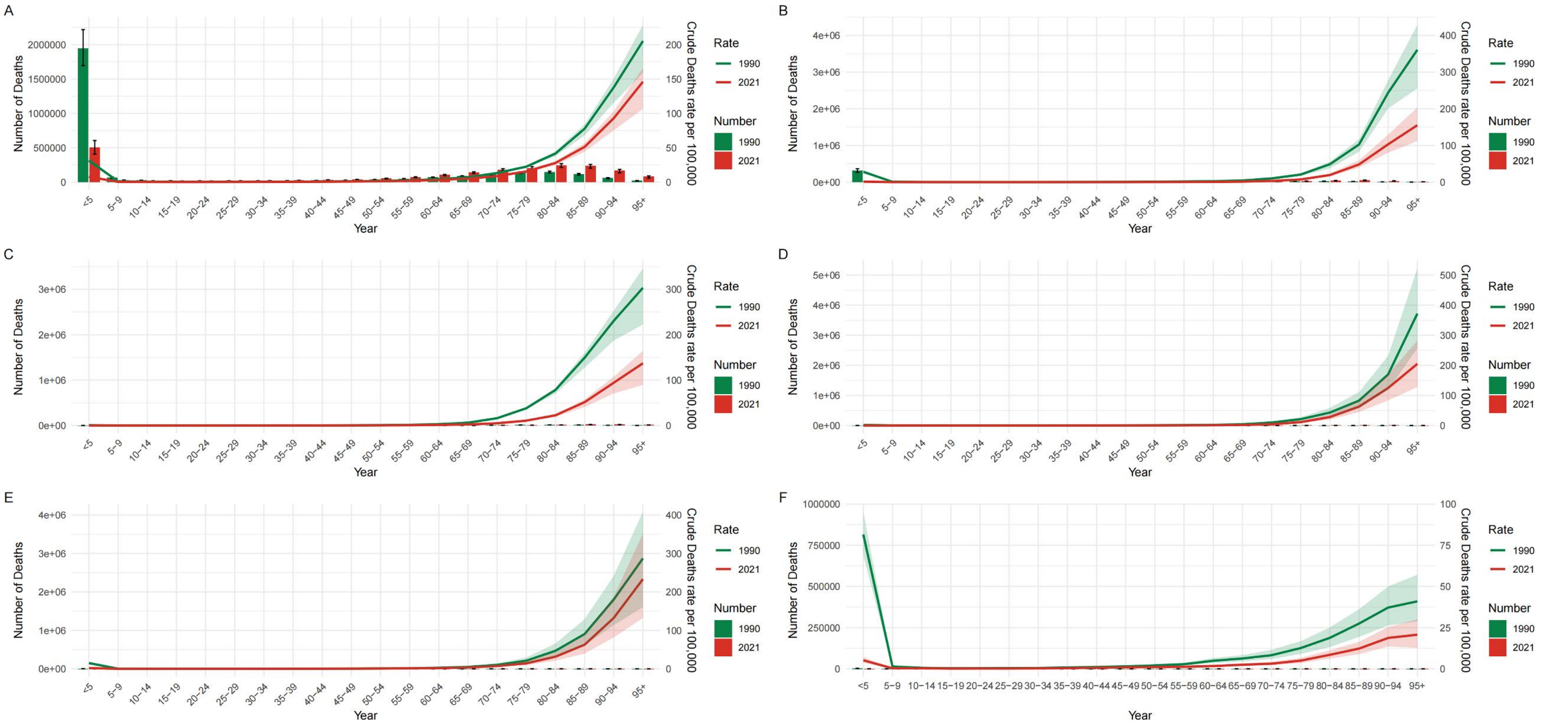

Figure S1. Death numbers and crude death rates of lower respiratory infections by age group globally and in Five East Asian Countries, 1990 versus 2021. (A) Global; (B) China; (C) Japan; (D) Republic of Korea; (E) Democratic People's Republic of Korea; (F) Mongolia.

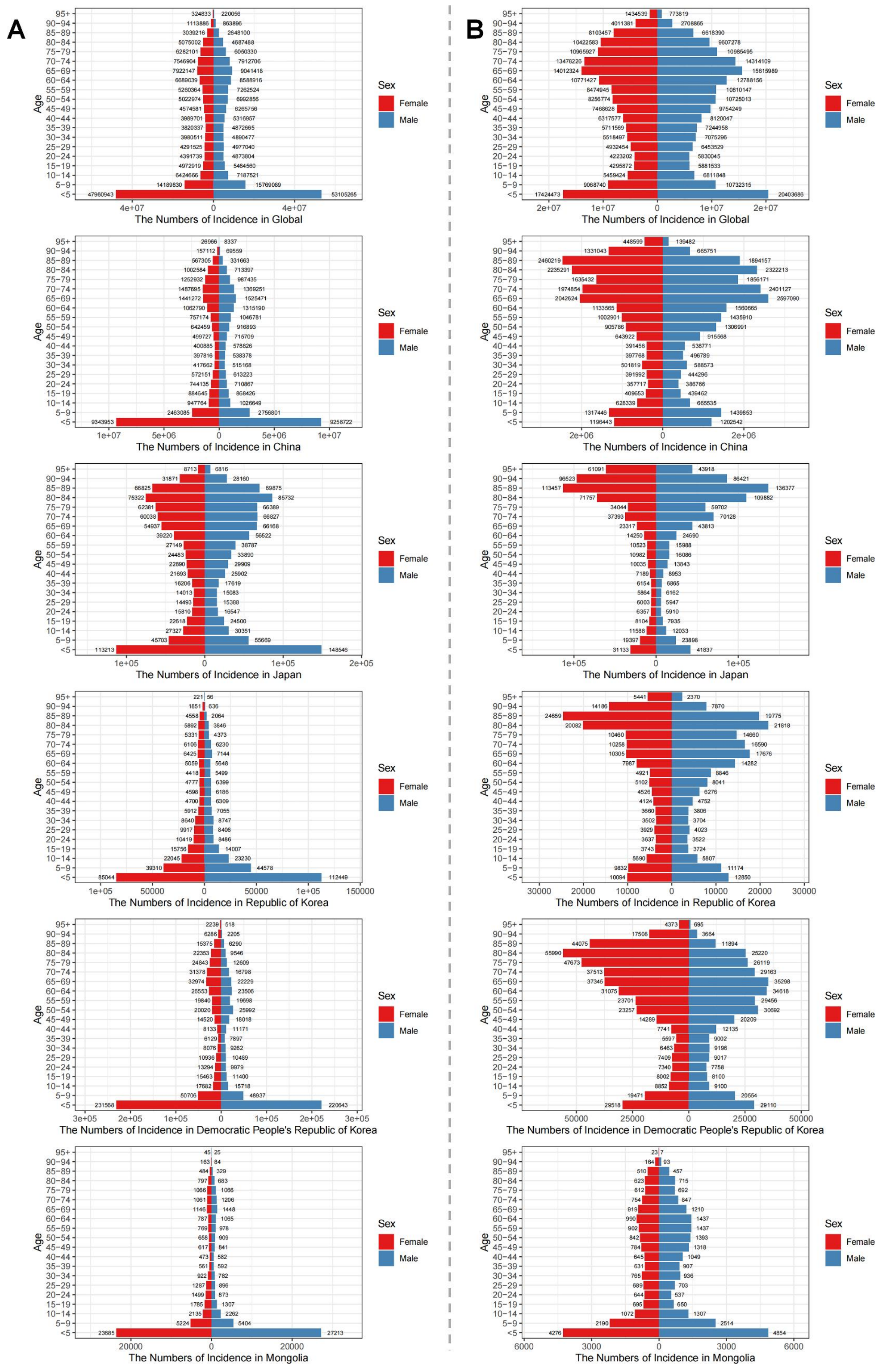

Figure S2. Age-sex distribution of incidence numbers of lower respiratory infections globally and in Five East Asian Countries in 1990 and 2021. (A) Incidence numbers in 1990; (B) Incidence numbers in 2021.

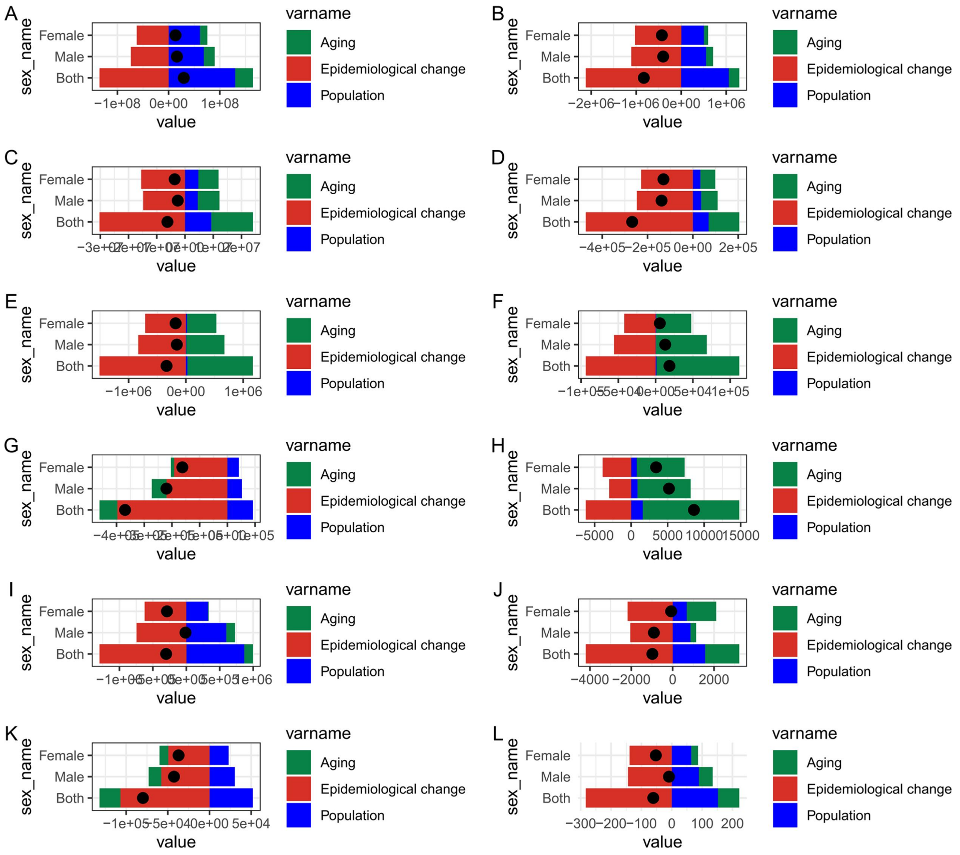

Figure S3. Decomposition analysis of changes in incidence and deaths due to lower respiratory infections attributable to aging, population growth, and epidemiological change globally and in Five East Asian Countries by sex. (A-B) Global; (C-D) China; (E-F) Japan; (G-H) Republic of Korea; (I-J) Democratic People's Republic of Korea; (K-L) Mongolia.

Table S1. Incidence, prevalence, deaths, and DALYs for lower respiratory infections globally and in Five East Asian Countries, 1990 versus 2021. DALYs: Disability-Adjusted Life Years; UI: Uncertainty Interval; ASRs: Age-standardized Rates; CI: Confidence Interval.

Location	Measure	1990		2021		Percentage change in the ASRs per 100,000 people
		All-ages cases	Age-standardized rate per 100,000 people	All-ages cases	Age-standardized rate per 100,000 people	
		n(95% UI)	n(95% UI)	n(95% UI)	n(95% UI)	%(95% CI)
Global	Incidence	313,864,642(294,055,265-333,297,862)	6,373.172(5,993.512-6,746.037)	343,606,787(325,214,314-363,517,285)	4,283.613(4,057.026-4,524.887)	-32.8 (-34.8,-30.6)
	Prevalence	6,952,663(6,531,632-7,404,351)	140.557(132.939-148.867)	7,569,580(7,198,813-8,005,307)	94.424(89.783-99.839)	-32.8 (-34.9,-30.7)
	Deaths	3,013,349(2,744,313-3,291,759)	61.814(56.657-66.737)	2,183,001(1,979,915-2,360,084)	28.671(25.924-31.066)	-53.6 (-57.6,-49.4)
	DALYs	204,174,057(180,683,529-229,006,748)	3,472.901(3,090.707-3,872.106)	82,534,841(72,611,990-93,402,507)	1,168.803(1,016.961-1,336.948)	-66.3 (-70.5,-61.5)
China	Incidence	50,936,862(47,531,532-54,596,483)	5,481.131(5,149.055-5,836.346)	44,704,579(41,780,823-47,783,965)	2,853.807(2,663.944-3,067.554)	-47.9 (-50.3,-45.4)
	Prevalence	1,141,642(1,068,391-1,221,122)	122.234(115.101-129.717)	1,002,599(941,423-1,072,748)	64.318(60.241-69.067)	-47.4 (-49.8,-44.6)
	Death	474,883(414,631-532,255)	60.651(52.956-66.662)	206,930(171,261-251,990)	14.031(11.684-17.003)	-76.9 (-80.9,-71.2)

	s					
	DALYs	32,576,310(28,139,744-37,498,333)	3,128.386(2,724.105-3,579.574)	4,106,779(3,504,141-4,808,453)	347.665(301.284-402.941)	-88.9 (-91.1,-86.3)
Japan	Incidence	1,663,583(1,567,698-1,772,285)	1,402.089(1,309.380-1,513.349)	1,325,551(1,255,268-1,410,226)	608.720(562.352-659.169)	-56.6 (-57.8,-55.3)
	Prevalence	36,140(34,103-38,556)	30.940(28.754-33.388)	27,281(25,859-28,864)	13.794(12.620-15.138)	-55.4 (-57.1,-53.8)
	Deaths	61,024(55,029-64,218)	41.079(36.610-43.417)	79,581(63,674-88,419)	13.889(11.636-15.174)	-66.2 (-68.5,-64.5)
	DALYs	987,952(917,182-1,029,124)	668.021(619.197-696.501)	946,418(790,104-1,034,087)	215.584(190.999-230.018)	-67.7 (-69.4,-66.4)
Republic of Korea	Incidence	532,326(484,952-585,397)	1,505.928(1,382.288-1,640.938)	357,704(333,514-382,483)	666.992(610.602-726.717)	-55.7 (-58.4,-52.6)
	Prevalence	12,308(11,245-13,565)	34.192(31.461-37.354)	7,665(7,147-8,251)	14.873(13.587-16.309)	-56.5 (-59.2,-53.4)
	Deaths	5,516(4,793-6,551)	28.642(24.048-35.231)	14,118(10,891-16,733)	15.884(12.272-18.856)	-44.5 (-58,-33.8)
	DALYs	172,805(154,095-195,891)	609.382(537.337-712.294)	192,128(151,558-224,853)	221.877(177.998-257.749)	-63.6 (-70.7,-57)
Democratic		1,081,272(999,927-1	5,865.664(5,454.967-	798,191(741,990-86	3,004.085(2,792.982-	-48.8 (-52.1,-45.3)

People's Republic of Korea	Incidence	,170,058)	6,312.389)	4,297)	3,239.123)	
	Prevalence	24,409(22,625-26,402)	132.330(123.374-142.422)	17,537(16,281-18,863)	66.143(61.348-71.084)	-50 (-53.3,-46.8)
	Deaths	6,789(5,378-8,558)	43.941(34.574-54.742)	5,808(4,390-7,537)	23.292(17.385-29.936)	-47 (-60.1,-30.3)
	DALYs	401,819(308,633-520,079)	1,833.681(1,428.712-2,334.211)	139,207(105,230-180,724)	589.166(430.412-769.768)	-67.9 (-76.9,-54.2)
Mongolia	Incidence	93,711(85,672-102,256)	4,204.509(3,890.223-4,508.854)	41,793(39,047-44,849)	1,486.428(1,397.817-1,575.153)	-64.6 (-66.8,-62.2)
	Prevalence	2,087(1,921-2,271)	93.200(86.627-99.719)	894(837-960)	31.502(29.670-33.597)	-66.2 (-68.3,-64.1)
	Deaths	3,030(2,586-3,494)	101.526(87.683-115.272)	420(342-508)	14.434(11.984-17.167)	-85.8 (-88.5,-82.8)
	DALYs	257,990(217,735-299,176)	7,874.075(6,715.524-9,077.785)	26,949(21,166-34,100)	775.970(615.843-972.731)	-90.1 (-92.4,-87.2)

Table S2. Decomposition analysis of changes in incidence and deaths for lower respiratory infections globally and in Five East Asian Countries by sex, attributed to aging, population growth, and epidemiological changes, from 1990 to 2021.

UI: Uncertainty Interval.DALYs: Disability-Adjusted Life Years

Location	Measure	Sex	Overll difference	Change due to Population-level determinants (% contribute to the total changes)		
				Aging	Population	Epidemiological change
Global	Incidence	Both	29,742,145.30	34,407,169.32	130,236,552.83	-134,901,576.84
				-115.68%	-437.89%	(-453.57%)
		Male	16,263,344.05	20,828,152.34	68,950,716.66	-73,515,524.95
				-128.07%	-423.96%	(-452.03%)
		Female	13,478,801.25	14,511,247.37	61,276,660.50	-62,309,106.62
				-107.66%	-454.62%	(-462.27%)
	Deaths	Both	-830,348.15	230,531.08	1,058,361.41	-2,119,240.63
				(-27.76%)	(-127.46%)	-255.22%
		Male	-401,121.74	153,501.31	554,125.03	-1,108,748.08
				(-38.27%)	(-138.14%)	-276.41%
		Female	-429,226.41	93,499.29	504,813.79	-1,027,539.49
				(-21.78%)	(-117.61%)	-239.39%
China	Incidence	Both	-6,232,282.48	14,773,563.61	9,272,714.80	-30,278,560.89
				(-237.05%)	(-148.79%)	-485.83%
		Male	-2,569,038.24	7,663,396.71	4,548,737.91	-14,781,172.86
				(-298.30%)	(-177.06%)	-575.36%
		Female	-3,663,244.24	7,125,193.30	4,726,104.59	-15,514,542.13
				(-194.51%)	(-129.01%)	-423.52%
	Deaths	Both	-267,953.13	135,417.49	69,003.31	-472,373.93

				(-50.54%)	(-25.75%)	-176.29%
		Male	-138,161.38	73,008.60	36,051.71	-247,221.69
				(-52.84%)	(-26.09%)	-178.94%
		Female	-129,791.75	65,374.99	32,894.51	-228,061.25
				(-50.37%)	(-25.34%)	-175.71%
Japan	Incidence	Both	-338,032.16	1,145,661.81	24,687.42	-1,508,381.39
				(-338.92%)	(-7.30%)	-446.22%
		Male	-158,289.61	669,123.03	5,295.74	-832,708.39
				(-422.72%)	(-3.35%)	-526.07%
		Female	-179,742.54	513,821.36	17,750.45	-711,314.35
				(-285.87%)	(-9.88%)	-395.74%
	Deaths	Both	18,557.04	110,862.69	1,292.78	-93,598.44
				-597.42%	-6.97%	(-504.38%)
		Male	12,876.10	68,295.00	308.197	-55,727.10
				-530.40%	-2.39%	(-432.79%)
		Female	5,680.94	46,906.47	818.305	-42,043.84
				-825.68%	-14.40%	(-740.09%)
Republic of Korea	Incidence	Both	-369,207.38	-64,410.32	92,143.35	-396,940.41
				-17.45%	(-24.96%)	-107.51%
		Male	-219,812.78	-53,666.07	52,587.64	-218,734.35
				-24.41%	(-23.92%)	-99.51%
		Female	-162,473.38	-11,956.00	41,373.32	-191,890.71
				-7.36%	(-25.46%)	-118.11%
	Deaths	Both	8,602.28	13,196.37	1,606.54	-6,200.63
				-153.41%	-18.68%	(-72.08%)

		Male	5,181.40	7,299.96	858.195	-2,976.76
				-140.89%	-16.56%	(-57.45%)
		Female	3,420.89	6,548.38	770.566	-3,898.05
				-191.42%	-22.53%	(-113.95%)
Democratic People's Republic of Korea	Incidence	Both	-301,726.40	130,137.35	865,968.31	-1,297,832.06
				(-43.13%)	(-287.00%)	-430.14%
		Male	-12,777.80	133,578.79	595,576.19	-741,932.79
				(-1045.40%)	(-4661.02%)	-5806.42%
		Female	-289,119.15	7,880.32	325,083.39	-622,082.85
				(-2.73%)	(-112.44%)	-215.16%
	Deaths	Both	-981.38	1,651.96	1,563.69	-4,197.02
				(-168.33%)	(-159.34%)	-427.67%
		Male	-907.99	272.226	862.175	-2,042.39
				(-29.98%)	(-94.95%)	-224.94%
		Female	-73.39	1,422.58	681.937	-2,177.90
				(-1938.38%)	(-929.20%)	-2967.57%
Mongolia	Incidence	Both	-79,839.75	-25,079.67	52,077.19	-106,837.26
				-31.41%	(-65.23%)	-133.81%
		Male	-42,583.46	-15,196.10	30,348.03	-57,735.40
				-35.69%	(-71.27%)	-135.58%
		Female	-37,085.28	-10,516.51	22,906.81	-49,475.58
				-28.36%	(-61.77%)	-133.41%
	Deaths	Both	-60.84	69.884	152.368	-283.09
				(-114.87%)	(-250.44%)	-465.30%
		Male	-9.18	45.72	89.083	-143.985

				(-498.04%)	(-970.40%)	-1568.46%
		Female	-52.41	21.97	64.207	-138.587
				(-41.92%)	(-122.51%)	-264.43%

Table S3. Optimal ARIMA models for forecasting incidence and deaths of lower respiratory infections globally and in Five East Asian Countries. ARIMA: Autoregressive Integrated Moving Average; AIC: Akaike Information Criterion; BIC: Bayesian Information Criterion; Ljung_box: Ljung–Box test (P-value) for residual autocorrelation.

Location	Measure	Model(p.d.q)	AIC	BIC	Ljung_box
Global	Incidence	0,1,0	352.262	355.13	1
	Deaths	0,1,0	71.392	74.26	0.577
China	Incidence	1,1,0	342.405	346.707	0.996
	Deaths	0,2,0	58.179	59.581	0.884
Japan	Incidence	1,1,0	264.977	269.279	0.982
	Deaths	0,1,1	99.321	103.623	0.214
Republic of Korea	Incidence	0,2,0	247.864	249.265	1
	Deaths	0,2,0	43.355	44.756	0.975
Democratic People's Republic of Korea	Incidence	1,1,0	324.273	328.575	1
	Deaths	0,2,1	50.698	53.5	0.783
Mongolia	Incidence	0,1,0	326.923	329.791	0.985
	Deaths	0,2,1	71.909	74.712	0.808
